# C-Reactive Protein/Albumin Ratio Is an Independent Risk Factor for Recurrence and Survival Following Curative Resection of Stage I–III Colorectal Cancer in Older Patients

**DOI:** 10.1245/s10434-024-14961-2

**Published:** 2024-01-27

**Authors:** Tomoaki Bekki, Manabu Shimomura, Minoru Hattori, Saki Sato, Atsuhiro Watanabe, Sho Ishikawa, Kouki Imaoka, Kosuke Ono, Keiso Matsubara, Tetsuya Mochizuki, Shintaro Akabane, Takuya Yano, Hideki Ohdan

**Affiliations:** 1 Department of Gastroenterological and Transplant Surgery, Graduate School of Biomedical and Health Sciences, Hiroshima University, Hiroshima, Japan; 2 Advanced Medical Skills Training Center, Institute of Biomedical and Health Science, Hiroshima University, Hiroshima, Japan

**Keywords:** Colorectal cancer, Older patients, Curative resection, C-reactive protein/albumin ratio, Nutritional and inflammation-based indices

## Abstract

**Background:**

The number of older patients with cancer has increased, and colorectal cancer is expected to be affected by this trend. This study aimed to compare prognostic factors, including nutritional and inflammation-based indices, between patients aged ≥ 70 and < 70 years following curative resection of stage I–III colorectal cancer.

**Patients and Methods:**

This study included 560 patients with stage I–III colorectal cancer who underwent curative resection between May 2010 and June 2018. A retrospective analysis was performed to identify prognosis-associated variables in patients aged ≥ 70 and < 70 years.

**Results:**

Preoperative low body mass index, high C-reactive protein/albumin ratio, and comorbidities were mainly associated with poor prognosis in patients aged ≥ 70 years. Tumor factors were associated with a poor prognosis in patients aged < 70 years. The C-reactive protein/albumin ratio was independently associated with poor overall survival and recurrence-free survival in those aged ≥ 70 years. The time-dependent area under the curve for the C-reactive protein/albumin ratio was superior to those of other nutritional and inflammation-based indices in most postoperative observation periods in patients aged ≥ 70 years.

**Conclusions:**

Tumor factors were associated with a poor prognosis in patients aged < 70 years. In addition to lymph node metastasis, preoperative statuses were associated with poor prognosis in patients aged ≥ 70 years. Specifically, the preoperative C-reactive protein/albumin ratio was independently associated with long-term prognosis in patients aged ≥ 70 years with stage I–III colorectal cancer after curative resection.

**Supplementary Information:**

The online version contains supplementary material available at 10.1245/s10434-024-14961-2.

Colorectal cancer (CRC) is the most frequently diagnosed gastrointestinal cancer and the second most common new cause of cancer-related death worldwide.^1^ The number of older patients with cancer is increasing, and by 2030, approximately 70% of all new cancer cases will be diagnosed in individuals aged ≥ 65 years.^2^ Predictably, new CRC cases will be significantly influenced by aging, and the number of older patients with CRC is predicted to increase.

Older patients have various comorbidities, low physical fitness (such as frailty), and age-related declines in organ function and immunity.^3-5^ Compared with younger patients, older patients are considered at a higher risk of developing postoperative complications and require more careful perioperative management. Multi-interventional enhanced recovery after surgery programs^6^ are essential to reducing postoperative complications and achieving early recovery. In addition, identifying novel prognostic factors and biomarkers for older patients and selecting appropriate treatment strategies, including the implementation of postoperative adjuvant chemotherapy, are extremely important.

The central role of systemic inflammatory responses in cancer progression has been reported,^7^ and influences tumor prognosis by providing a suitable environment for tumor progression.^8^ On the basis of this concept, several simple nutritional and inflammation-based indices calculated from serum parameters, including neutrophils, lymphocytes, platelets, albumin (Alb), and C-reactive protein (CRP), have been developed and are reported to be related to CRC prognosis.^9-13^ One of these factors, the C-reactive protein/albumin ratio (CAR), can sensitively detect systemic inflammation because it is calculated from serum CRP and Alb levels, which are influenced by liver synthesis in the presence of inflammation.^14^ CAR is affected by age and tends to be higher in older people.^12,15,16^ However, a few studies have reported the effect of CAR on the prognosis of older patients with CRC.^17^

This retrospective study investigated prognostic factors, including preoperative nutritional and inflammation-based indices, and compared factors that affect long-term prognosis between patients aged < 70 and ≥ 70 years who underwent curative resection for stage I–III CRC.

## Patients and Methods

### Study Population

**Fig. 1:**
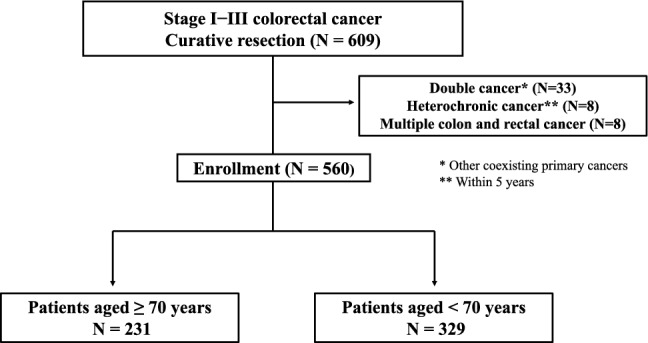
Flowchart of the study design

This retrospective study evaluated 609 patients with stage I–III CRC who underwent R0 resection between May 2010 and June 2018 at the Hiroshima University Hospital. Patients with other primary malignancies in the same period, heterochronic CRC within 5 years, or coexisting colon and rectal cancers were excluded. Finally, 560 patients who underwent R0 resection were enrolled in this study (Fig 1).

This study was conducted in accordance with the guidelines of the Declaration of Helsinki (Fortaleza, Brazil, October 2013) and approved by the Institutional Review Board of Hiroshima University Hospital (Approval No. E-744-4).

### Definition of Nutritional and Inflammation-Based Indices

Nutritional and inflammation-based indices were calculated by preoperative blood examinations. CAR was calculated as the serum CRP level (mg/dL)/serum Alb level (g/dL). Neutrophil/lymphocyte ratio (NLR) was calculated as relative neutrophil (%)/relative lymphocyte (%). Platelet/lymphocyte ratio (PLR) was calculated as the absolute number of platelets/lymphocytes. Prognostic nutritional index (PNI) was calculated as 10 × Alb level (g/dL) + 0.005 × total lymphocyte count (per mm^3^).^18^

### Treatment and Follow-Up

Follow-up blood examinations to identify tumor markers were performed every 3–6 months 5 years after surgery. Simple or enhanced abdominal computed tomography was performed to rule out recurrence in 6–12 months, and colonoscopy was performed in the first, third, and fifth years after surgery. Patients with high-risk stage II or III CRC underwent postoperative adjuvant chemotherapy (ACT). Postoperative ACT for older patients or those with severe comorbidities was performed at the discretion of the primary surgeon on the basis of their general condition.

### Statistical Analysis

Continuous variables are presented as medians and ranges. Nominal variables are expressed as numbers (%). Nonparametric quantitative data were analyzed using the Mann–Whitney *U*-test. The chi-squared test or Fisher’s exact test were performed to determine the relationships among nominal variables. The cutoff values for nutritional and inflammation-based indices were set using receiver operating characteristic (ROC) curve analysis. The Kaplan–Meier method was used to analyze overall survival (OS) and recurrence-free survival (RFS), and the log-rank test was used to compare different groups. Multivariate analyses were performed to assess the factors influencing OS and RFS using the Cox regression model.

To overcome the bias caused by different distributions of covariates among patients from the high and low-CAR groups, propensity score-matched (PSM) analysis was performed using a multiple logistic regression model based on the clinicopathological variables. PSM analysis was performed according to baseline characteristics, such as age, American Society of Anesthesiologists Physical Status (ASA-PS), tumor markers, nutritional or inflammation-based indices, surgery-related factors, and tumor depth, which were variables that differed significantly (*P* values < 0.05). The prognostic capabilities of the CAR, PNI, NLR, and PLR were compared using time-dependent ROC curves and the area under the curve (AUC). Harrell’s concordance index (C-index) was calculated for each model to determine which index had a predictive association with the endpoints. Bootstrapping method was used to calculate 95% confidence intervals (CI). *P* values < 0.05 were considered statistically significant. Calculations were performed using JMP v17 (SAS Institute, Cary, NC, USA) and EZR Ver 1.61 (Saitama Medical Center, Jichi Medical University, Saitama, Japan).^19^

## Results

### Patient Characteristics of Patients with CRC Aged ≥ 70 and < 70 Years

Table 1 summarizes the preoperative characteristics of patients aged ≥ 70 and < 70 years with stage I–III CRC who underwent R0 resection. Overall, 231 (41.3%) patients were ≥ 70 years old and 329 (58.7%) were < 70 years old. Compared with patients aged < 70 years, those aged ≥ 70 years included more patients with ASA-PS ≥ 3 (*P* = 0.0001) and more patients with hypertension (HTN; *P* < 0.0001), diabetes mellitus (DM; *P* = 0.0022), and cardiovascular disease (*P* < 0.0001), and more frequently used antiplatelet or coagulation agents (*P* < 0.0001). Among the nutritional and inflammation-based indices, CAR was higher in patients aged ≥ 70 years (*P* = 0.0036) than in those aged < 70 years. PNI was lower (*P* < 0.0001) and operative time was shorter in patients aged ≥ 70 years (*P* = 0.0004). In patients aged ≥ 70 years, the proportion of patients who underwent laparoscopic or robot-assisted surgery (*P* = 0.0471) and the implementation of postoperative ACT (*P* < 0.0001) were lower. Tumor localization was less frequent in the rectum (*P* < 0.0001). No significant differences were observed in tumor factors or postoperative complications between patients aged ≥ 70 years and aged < 70 years groups. A total of 447 patients were assessed for budding grades. The number of patients with budding grade > 1 was 118 (45.2%) in those aged < 70 years and 76 (40.9%) in those aged ≥ 70 years. Moreover, 281, 237, and 274 patients were screened for the presence of RAS, BRAF, and microsatellite instability (MSI) mutations, respectively. Of these patients, 122 (43.4%) had RAS mutations, 14 (5.9%) had BRAF mutations, and 25 (9.1%) had MSI-high. No differences in biological markers according to age were noted.

**Table 1:** Comparison of patient clinical characteristics with age

Variables	Age ≥ 70 group (*n* = 231)	Age < 70 group (*n* = 329)	*P* value
Sex (M/F)	137/94	207/122	0.3879
BMI (kg/m^2^)	22.4 (14.2–33.4)	22.7 (14.7–49.8)	0.3939
ASA-PS ≥ 3	23 (10.0%)	8 (2.4%)	**0.0001**
HTN (+)	113 (48.9%)	100 (30.4%)	**< 0.0001**
DM (+)	60 (26.0%)	51 (15.5%)	**0.0022**
DL (+)	77 (33.3%)	103 (31.3%)	0.6136
Cardiovascular disease (+)	51 (22.1%)	19 (5.8%)	**< 0.0001**
Ventilatory disease (+)	23 (10.0%)	22 (6.7%)	0.1611
Medications: antiplatelet or coagulation agents	50 (21.7%)	20 (6.1%)	**< 0.0001**
CEA (ng/mL)	3.0 (0.6–231.1)	2.7 (0.5–351.1)	0.0872
CA19-9 (U/mL)	7.0 (1.0–1413)	6.0 (0.0–9029)	0.4689
PNI	49.9 (19.9–63.3)	52.3 (31.4–64.3)	**< 0.0001**
CAR	0.024 (0.0–13.8)	0.017 (0.0–2.11)	**0.0036**
NLR	2.3 (0.7–97.0)	2.2 (0.7–24.4)	0.2546
PLR	136.8 (29.0–1867)	135.8 (38.8–3549)	0.8498
Open/laparoscopic or robot-assisted	51/180	51/278	**0.0471**
Operative time (min)	268 (99–559)	292 (125–813)	**0.0004**
Blood loss (mL)	65 (5–3100)	54 (0–3407)	0.1340
Intraoperative blood transfusion (+)	13 (5.6%)	10 (3.1%)	0.1343
Tumor localization: colon/rectum	154/77	155/174	**< 0.0001**
pT ≥ 4	11 (4.8%)	17 (5.2%)	0.8285
pN (+)	65 (28.1%)	97 (29.5%)	0.7297
Histology: other than differentiated carcinoma	35 (15.2%)	36 (11.0%)	0.1520
Vascular invasion (+)	147 (63.6%)	209 (63.7%)	0.9839
Postoperative chemotherapy (+)	45 (19.5%)	134 (40.7%)	**< 0.0001**
Postoperative complications CD ≥ 3 (+)	15 (6.5%)	28 (8.5%)	0.3775

The variables in bold are statistically significant (*P* < 0.05). Continuous variables are expressed as medians (ranges). Qualitative variables are expressed as numbers (%).

*M* male, *F* female, *BMI* body mass index, *ASA-PS* American Society of Anesthesiologists Physical Status, *HTN* hypertension, *DM* diabetes mellitus, *DL* dyslipidemia, *CEA* carcinoembryonic antigen, *CA19-9* carbohydrate antigen 19-9, *PNI* prognostic nutrition index, *CAR* C-reactive protein/albumin ratio, *NLR* neutrophil/lymphocyte ratio, *PLR* platelet/lymphocyte ratio, *CD* Clavien–Dindo

### Comparison of Univariate and Multivariate Analyses of Prognostic Factors for OS and RFS in Patients Aged ≥ 70 and < 70 Years

Table 2 summarizes the results of the univariate and multivariate analyses of prognostic factors for OS in patients aged ≥ 70 years. In the univariate analysis, statistically significant prognostic factors for poor OS were body mass index (BMI) < 25 (kg/m^2^; *P* = 0.0253), ASA-PS ≥ 3 (*P* = 0.0201), DM (*P* = 0.0109), carcinoembryonic antigen (CEA) > 5 (ng/mL; *P* = 0.0022), carbohydrate antigen 19-9 (CA19-9) > 37 (U/mL; *P* = 0.0178), CAR ≥ 0.03 (*P* = 0.0042), NLR > 3.0 (*P* = 0.0198), and pathological positivity of lymph node metastasis (*P* = 0.0016). In the multivariate analysis, the following four factors were identified as prognostic factors for poor OS in patients aged ≥ 70 years: BMI < 25 (kg/m^2^) [hazard ratio (HR) 5.230; 95% CI 1.20–22.8, *P* = 0.0277], DM (HR 2.487; 95% CI 1.10–5.62, *P* = 0.0286), CAR ≥ 0.03 (HR 2.385; 95% CI 1.03–5.55, *P* = 0.0435), and pathological positivity of lymph node metastasis (HR 2.892; 95% CI 1.24–6.75, *P* = 0.0141).

**Table 2:** Univariate and multivariate analyses of prognostic factor for overall survival in patients aged ≥ 70 years

Variables		Univariate			Multivariate		
	*N* = 231	HR	95% CI	*P*-value	HR	95% CI	*P* value
Male	94	1.784	0.79–4.03	0.1647			
BMI (kg/m^2^) < 25	179	5.204	1.23–22.1	**0.0253**	5.230	1.20–22.8	**0.0277**
ASA-PS ≥ 3	23	3.176	1.20–8.42	**0.0201**	2.291	0.77–6.79	0.1350
HTN (+)	113	0.587	0.26–1.31	0.1930			
DM (+)	60	2.599	1.25–5.42	**0.0109**	2.487	1.10–5.62	**0.0286**
DL (+)	77	0.605	0.26–1.38	0.2327			
Cardiovascular disease (+)	51	1.423	0.62–3.24	0.4013			
Ventilatory disease (+)	23	0.666	0.16–2.81	0.5803			
Medications: antiplatelet or coagulation agents	50	0.903	0.37–2.22	0.8248			
CEA (ng/mL) > 5	65	3.154	1.51–6.59	**0.0022**	1.386	0.55–3.47	0.4854
CA19-9 (U/mL) > 37	16	3.243	1.22–8.58	**0.0178**	1.148	0.33–4.02	0.8290
PNI < 47	75	1.744	0.83–3.67	0.1421			
CAR ≥ 0.03	102	3.158	1.43–6.94	**0.0042**	2.385	1.03-5.55	**0.0435**
NLR > 3.0	71	2.455	1.16–5.20	**0.0198**	1.880	0.85-4.18	0.1209
PLR > 113	148	0.649	0.31–1.37	0.2553			
Laparoscopic/robot-assisted	180	0.808	0.34–1.90	0.6249			
Operative time (min) > 275	106	1.679	0.80–3.54	0.1734			
Blood loss (mL) ≥ 60	128	0.975	0.47–1.03	0.9452			
Intraoperative blood transfusion (+)	13	2.506	0.74–8.44	0.1382			
Tumor localization: rectum	77	1.563	0.74–3.30	0.2411			
pT ≥ 4	11	2.482	0.58–10.6	0.2197			
pN (+)	65	3.267	1.57–6.80	**0.0016**	2.892	1.24–6.75	**0.0141**
Histology: other than differentiated carcinoma	35	1.348	0.51–3.54	0.5444			
Vascular invasion (+)	147	1.219	0.54–2.77	0.6354			
Postoperative chemotherapy (+)	45	0.769	0.31–1.90	0.5686			
Postoperative complications CD ≥ 3 (+)	15	1.357	0.40–4.57	0.6220			

The variables in bold are statistically significant (*P* < 0.05).

*HR* hazard ratio, *CI* confidence interval, *BMI* body mass index, *ASA-PS* American Society of Anesthesiologists Physical Status, *HTN* hypertension, *DM* diabetes mellitus, *DL* dyslipidemia, *CEA* carcinoembryonic antigen, *CA19-9* carbohydrate antigen 19-9, *PNI* prognostic nutrition index, *CAR* C-reactive protein/albumin ratio, *NLR* neutrophil/lymphocyte ratio, *PLR* platelet/lymphocyte ratio, *CD* Clavien–Dindo

Table 3 summarizes the results of the univariate and multivariate analyses of prognostic factors for RFS in patients aged ≥ 70 years. In the univariate analysis, statistically significant prognostic factors for poor RFS were ASA-PS ≥ 3 (*P* = 0.0086), DM (*P* = 0.0147), CEA > 5 (ng/mL; *P* = 0.0006), CA19-9 > 37 (U/mL; *P* = 0.0004), CAR ≥ 0.03 (*P* = 0.0049), pT ≥ 4 (*P* = 0.0194), and pathological positivity of lymph node metastasis (*P* = 0.0003). In the multivariate analysis, the following three factors were identified as prognostic factors of poor RFS in patients aged ≥ 70 years: ASA-PS ≥ 3 (HR 3.480; 95% CI 1.35–8.95, *P* = 0.0096), CAR ≥ 0.03 (HR 2.075; 95% CI 1.04–4.13, *P* = 0.0376), and pathological positivity of lymph node metastasis (HR 2.767; 95% CI 1.37–5.57, *P* = 0.0043).

**Table 3:** Univariate and multivariate analyses of prognostic factors for recurrence-free survival in patients aged ≥ 70 years

Variables		Univariate			Multivariate		
	*N* = 231	HR	95% CI	*P*-value	HR	95% CI	*P* value
Male	94	1.632	0.83–3.20	0.1546			
BMI (kg/m^2^) < 25	179	2.035	0.85–4.87	0.1109			
ASA-PS ≥ 3	23	3.001	1.32–6.81	**0.0086**	3.480	1.35–8.95	**0.0096**
HTN (+)	113	0.681	0.36–1.30	0.2455			
DM (+)	60	2.177	1.17–4.06	**0.0147**	1.349	0.66–2.77	0.4146
DL (+)	77	1.048	0.55–1.98	0.8848			
Cardiovascular disease (+)	51	0.970	0.45–2.07	0.9380			
Ventilatory disease (+)	23	0.662	0.20–2.15	0.4918			
Medications: antiplatelet or coagulation agents	50	1.085	0.53–2.23	0.8245			
CEA (ng/mL) > 5	65	2.940	1.59–5.44	**0.0006**	1.318	0.61–2.87	0.4866
CA19-9 (U/mL) > 37	16	4.557	1.98–10.5	**0.0004**	1.656	0.62–4.46	0.3177
PNI < 47	75	1.481	0.78–2.81	0.2296			
CAR ≥ 0.03	102	2.499	1.32–4.73	**0.0049**	2.075	1.04–4.13	**0.0376**
NLR > 3.0	71	1.785	0.95–3.37	0.0741			
PLR > 113	148	1.092	0.57–2.09	0.7920			
Laparoscopic/robot-assisted	180	0.721	0.36–1.44	0.3556			
Operative time (min) > 275	106	1.586	0.85–2.97	0.1501			
Blood loss (mL) ≥ 60	128	1.026	0.55–1.91	0.9342			
Intraoperative Blood transfusion (+)	13	2.237	0.78–6.42	0.1344			
Tumor localization: rectum	77	1.337	0.71–2.51	0.3660			
pT ≥ 4	11	3.466	1.22–9.82	**0.0194**	2.118	0.66–6.78	0.2060
pN (+)	65	3.180	1.71–5.92	**0.0003**	2.767	1.37–5.57	**0.0043**
Histology: other than differentiated carcinoma	35	0.835	0.33–2.14	0.7072			
Vascular invasion (+)	147	1.602	0.78–3.27	0.1962			
Postoperative chemotherapy (+)	45	1.234	0.62–2.47	0.5527			
Postoperative complications CD ≥ 3 (+)	15	1.344	0.47–3.83	0.5803			

The variables in bold are statistically significant (*P* < 0.05).

*HR* hazard ratio, *CI* confidence interval, *BMI* body mass index, *ASA-PS* American Society of Anesthesiologists Physical Status, *HTN* hypertension, *DM* diabetes mellitus, *DL* dyslipidemia, *CEA* carcinoembryonic antigen, *CA19-9* carbohydrate antigen 19-9, *PNI* prognostic nutrition index, *CAR* C-reactive protein/albumin ratio, *NLR* neutrophil/lymphocyte ratio, *PLR* platelet/lymphocyte ratio, *CD* Clavien–Dindo

**Fig. 2:**
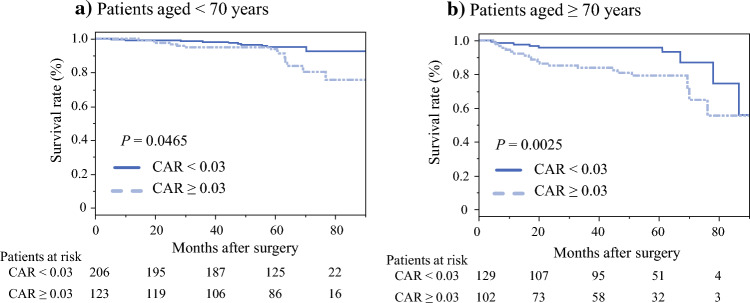
**a, b** Kaplan–Meier curves for overall survival in patients aged < 70 and ≥ 70 years used to compare the high and low-CAR groups

**Fig. 3:**
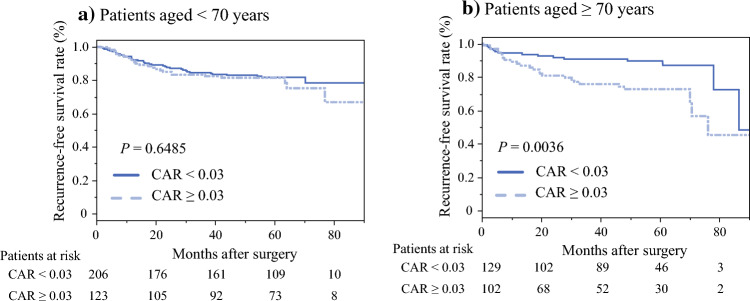
**a, b** Kaplan–Meier curves for recurrence-free survival in patients aged < 70 and ≥ 70 years used to compare the high and low-CAR groups

Table S1 summarizes the results of the univariate and multivariate analyses of prognostic factors for OS in patients aged < 70 years. In the univariate analysis, statistically significant prognostic factors for poor OS were HTN (*P* = 0.0088), CEA > 5 (ng/mL; *P* = 0.0218), CA19-9 level > 37 (U/mL; *P* < 0.0001), pathological positivity of lymph node metastasis (*P* = 0.0103), positivity for vascular invasion (*P* = 0.0252), and implementation of postoperative ACT (*P* = 0.0235). In the multivariate analysis, CA19-9 > 37 (U/mL; HR 5.317; 95% CI 1.77–15.9 *P* = 0.0028) was the only prognostic factor of poor OS in patients aged < 70 years. Table S2 summarizes the results of the univariate and multivariate analyses of prognostic factors for RFS in patients aged < 70 years. In the univariate analysis, the statistically significant prognostic factors for poor RFS were CEA > 5 (ng/mL; *P* = 0.0004), CA19-9 > 37 (U/mL; *P* = 0.0068), NLR > 3.0 (*P* = 0.0436), surgical procedure (*P* = 0.0302), operative time > 275 (min; *P* = 0.0068), intraoperative blood loss ≥ 60 (mL; *P* = 0.0022), rectal cancer (*P* = 0.0013), pT ≥ 4 (*P* = 0.0226), pathological positivity of lymph node metastasis (*P* = 0.0002), positivity for vascular invasion (*P* = 0.0011), and implementation of postoperative ACT (*P* = 0.0002). In the multivariate analysis, the following two factors were identified as prognostic factors for poor RFS in patients aged < 70 years: rectal cancer (HR 1.863; 95% CI 1.02–3.40, *P* = 0.0423) and pathological positivity of lymph node metastasis (HR 2.147; 95% CI 1.06–4.36, *P* = 0.0347). Figures 2 and 3 summarize the Kaplan–Meier analysis showing OS and RFS using CAR. High CAR was associated with poor OS in aged < 70 years and ≥ 70 years groups (*P* = 0.0465, log-rank test; Fig. 2a, *P* = 0.0025, log-rank test; Fig. 2b). As for the RFS, high CAR was associated with poor RFS only in patients aged ≥ 70 years (*P* = 0.6485, log-rank test; Fig. 3a, *P* = 0.0036, log-rank test; Fig. 3b).

### Comparison of Backgrounds by Preoperative CAR Value

**Fig. 4:**
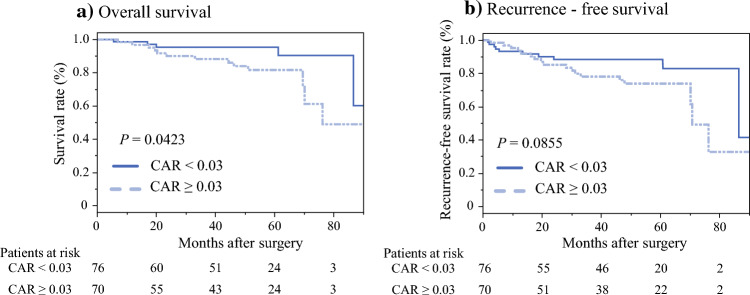
**a, b** Kaplan–Meier curves for overall survival and recurrence-free survival in patients aged ≥ 70 years after propensity score-matched analysis used to compare the high and low-CAR groups

Table 4 summarizes the characteristics of patients in the high and low-CAR groups. Compared with the low-CAR group, the high-CAR group included older patients (*P* = 0.0047), more patients with ASA-PS ≥ 3 (*P* = 0.0045), and more patients who underwent open surgery (*P* < 0.0001). Intraoperative blood loss (*P* < 0.0001), intraoperative blood transfusion (*P* = 0.0401), and the number of patients with pT ≥ 4 (*P* = 0.0001) were higher in the high-CAR group than in the low-CAR group. Tumor marker levels were higher in the high-CAR group than in the low-CAR group (CEA: *P* < 0.0001, CA19-9: *P* = 0.0006). Among the nutritional and inflammation-based indices, the PNI (*P* < 0.0001), NLR (*P* < 0.0001), and PLR (*P* < 0.0001) were significantly different between the two groups. After PSM analysis, no significant differences were found between the two groups. Figure 4 summarizes the Kaplan–Meier analysis showing the OS and RFS in patients aged ≥ 70 years after PSM using CAR. A high CAR was associated with a poor OS (*P* = 0.0423, log-rank test; Fig. 4a). Although not significant, a high CAR tended to show poor RFS (*P* = 0.0855, log-rank test; Fig. 4b).

**Table 4:** Characteristics of patients according to CAR value in the whole study series and the propensity score-matched study

	Whole study series			Propensity score-matched series		
	High-CAR group (*n* = 225)	Low-CAR group (*n* = 335)	*P* value	High-CAR group (*n* = 163)	Low-CAR group (*n* = 163)	*P* value
Age (year)	68 (30–94)	67 (23–93)	**0.0047**	68 (30–94)	68 (23–93)	0.7954
Sex (M/F)	135/90	209/126	0.5692	101/62	99/64	0.8201
BMI (kg/m^2^)	22.5 (14.2–49.8)	22.6 (14.7–33.4)	0.8467	22.9 (14.2–49.8)	23.2 (14.7–33.4)	0.1463
ASA-PS ≥ 3	20 (8.9%)	11 (3.3%)	**0.0045**	7 (4.3%)	9 (5.5%)	0.6081
HTN (+)	90 (40.0%)	123 (36.7%)	0.4326	64 (39.3%)	63 (38.7%)	0.9096
DM (+)	53 (23.6%)	58 (17.3%)	0.0693	36 (22.1%)	28 (17.2%)	0.2646
DL (+)	72 (32.0%)	108 (32.2%)	0.9527	57 (35.0%)	48 (29.5%)	0.2861
Cardiovascular disease (+)	31 (13.8%)	39 (11.6%)	0.4537	21 (12.9%)	24 (14.7%)	0.6300
Ventilatory disease (+)	23 (10.2%)	22 (6.6%)	0.1188	15 (9.2%)	11 (6.8%)	0.4135
Medications: antiplatelet or coagulation agents	32 (14.2%)	38 (11.3%)	0.3125	26 (16.0%)	23 (14.1%)	0.6420
CEA (ng/mL)	3.5 (0.6–351.1)	2.4 (0.5–240.8)	**< 0.0001**	3.0 (0.6–325)	2.6 (0.6–145)	0.1791
CA19-9 (U/mL)	8.0 (2.0–9029)	6.0 (0.0–80)	**0.0006**	6.0 (2.0–82)	6.0 (1.9–80)	0.9397
PNI	48.8 (19.9–63.9)	52.6 (33.2–64.3)	**< 0.0001**	50.2 (26.3–63.9)	50.4 (33.2–63.8)	0.9845
NLR	2.7 (0.7–97)	2.1 (0.7–24.4)	**< 0.0001**	2.4 (0.7–19.2)	2.3 (0.7–24.4)	0.7359
PLR	151.9 (40.4–3549)	129.7 (29.0–1197)	**< 0.0001**	137.8 (49.6–3549)	136.9 (29.0–1197)	0.5505
Open/laparoscopic or robot-assisted	63/162	39/296	**< 0.0001**	133/30	134/29	0.8856
Operative time (min)	293 (117–813)	278 (99–736)	0.3433	296 (125–813)	279 (153–736)	0.3212
Blood loss (mL)	80 (5–3407)	50 (0–3100)	**< 0.0001**	70 (5–1600)	62 (0–3100)	0.4082
Intraoperative blood transfusion (+)	14 (6.3%)	9 (2.7%)	**0.0401**	6 (3.7%)	3 (1.8%)	0.5017
Tumor localization: colon/rectum	130/95	179/156	0.3108	73 (44.8%)	83 (50.9%)	0.2676
pT ≥ 4	21 (9.3%)	7 (2.1%)	**0.0001**	4 (2.5%)	5 (3.1%)	1.0000
pN (+)	65 (28.9%)	97 (29.0%)	0.9865	48 (29.5%)	48 (29.5%)	1.0000
Histology: other than differentiated carcinoma	33 (14.7%)	38 (11.4%)	0.2492	19 (11.7%)	20 (12.4%)	0.8482
Vascular invasion (+)	146 (64.9%)	210 (62.9%)	0.6269	102 (62.6%)	111 (68.5%)	0.2598
Postoperative chemotherapy (+)	72 (32.0%)	107 (31.9%)	0.9881	50 (30.7%)	55 (33.7%)	0.5534
Postoperative complications CD ≥ 3 (+)	20 (8.9%)	23 (6.9%)	0.3780	13 (8.0%)	9 (5.5%)	0.3772

The variables in bold are statistically significant (P < 0.05). Continuous variables are expressed as medians (ranges). Qualitative variables are expressed as numbers (%).

*M* male, *F* female, *BMI* body mass index, *ASA-PS*, American Society of Anesthesiologists Physical Status, *HTN* hypertension, *DM* diabetes mellitus, *DL* dyslipidemia, *CEA* carcinoembryonic antigen, *CA19-9* carbohydrate antigen 19-9, *PNI* prognostic nutrition index, *CAR* C-reactive protein/albumin ratio, *NLR* neutrophil/lymphocyte ratio, *PLR* platelet/lymphocyte ratio, *CD* Clavien–Dindo

### Comparison of Nutritional and Inflammation-Based Indices for OS

**Fig. 5:**
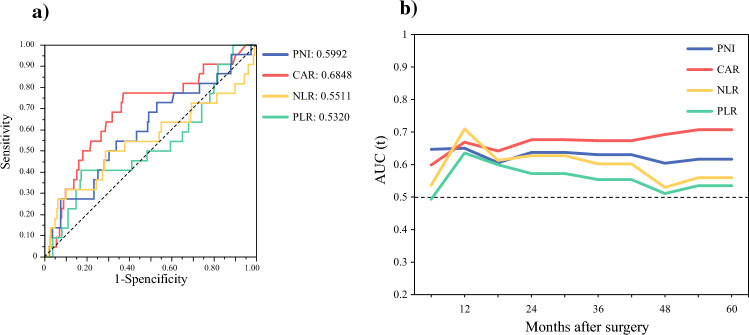
Comparison of each nutritional and inflammation-based index for OS in patients aged ≥ 70 years; **a** comparison of the areas under receiver operating curves at 5 years after surgery among the nutritional and inflammation-based indices, and **b** comparison of the time dependent areas under receiver operating curves between CAR and other indices

Figure 5 summarizes the comparison of each nutritional and inflammation-based index for OS in patients aged ≥ 70 years. Figure 5a shows a comparison of the ROC curve at 5 years after surgery. The AUC value of the CAR was 0.6848, which was higher than that of the other indices (PNI, 0.5992; NLR, 0.5511; PLR, 0.5320). A time-dependent ROC curve was used to calculate the AUC values at different timepoints. Harrell’s concordance index (C-index) values for CAR were superior to those for other nutritional and inflammation-based indices after surgery (CAR, 0.666; 95% CI 0.531–0.791; SE 0.065; PNI, 0.616; 95% CI 0.484–0.741; SE 0.066; NLR, 0.576; 95% CI 0.432–0.709; SE 0.073; PLR, 0.549; 95% CI 0.413–0.678; SE 0.067; Fig. 5b).

## Discussion

This retrospective study investigated and compared prognostic factors affecting long-term prognosis in patients aged < 70 and ≥ 70 years who underwent curative resection for stage I–III CRC. Interestingly, tumor factors, such as tumor markers and lymph node metastasis, were associated with poor long-term prognosis in younger patients. In addition to lymph node metastasis, preoperative factors, such as CAR, an inflammation-based marker, and comorbidities, were associated with poor long-term prognosis in older patients. Recently, the number of older patients with cancer has been increasing; in one study, 56.3% of patients with CRC were ≥ 70 years of age.^20^ The 231 patients in this study were ≥ 70 years old, accounting for 41.3% of the total patients. Generally, older patients have various comorbidities, malnutrition, and age-related declines in organ function and immunity.^3,4^ In recent years, frailty, which is defined as a state of reduced physiological reserve from pathological or iatrogenic stressors owing to age-related impairments,^21^ has received increasing attention. These factors increase perioperative complications and contribute to poor prognosis.^10,13,22-24^ In older patients, the preoperative evaluation of health-related priorities, realistic assessments of surgical risks, and individualized optimization strategies are important. In this study, statistical analysis revealed that high preoperative CAR was associated with poor OS and RFS in patients aged ≥ 70 years following curative resection of stage I–III CRC, and a high preoperative CAR was an independent poor prognostic factor for OS and RFS. Furthermore, a high preoperative CAR was found to be a better predictor of prognosis than other markers in a time-dependent ROC curve for OS. Recent studies have reported the association between systemic inflammation and various carcinomas.^7,8^ These indicators can be easily calculated and are expected to be useful for predicting preoperative prognosis in older patients.

In this study, CAR was a useful prognostic indicator for older patients following curative resection of stage I–III CRC. CAR was initially reported as an inflammation-based index to identify patients with serious illness.^25^ CAR is calculated on the basis of serum CRP and Alb levels, which are synthesized in hepatocytes. During the acute inflammatory phase, the serum concentration of CRP increases, whereas that of Alb is suppressed through the activation of inflammatory cytokines. Despite the weak correlation between nutritional intake and serum Alb levels, hypoalbuminemia has been reported to be associated with inflammation.^26^ In addition, inflammatory responses influence tumor development and prognosis by providing a suitable environment for tumors.^8^ On the basis of the above, CAR, calculated from two factors that exhibit antithetical responses during inflammation, can be considered a highly sensitive prognostic indicator of malignant tumors. This study showed that the CAR was affected by age and tumor factors, which is consistent with previous studies.^15,17^ In addition, the time-dependent ROC curve for CAR was superior to those for other inflammation-based indices in most postoperative observation periods in patients aged ≥ 70 years. Systemic inflammatory response is associated with patients’ nutritional, functional, and immunological statuses,^27^ and a high CAR has been reported to be a good predictor of poor survival outcomes in patients with CRC of various backgrounds, not only in older people.^12,13,16,17,28-31^

However, some studies have shown that CAR is not associated with poor prognosis in CRC.^32^ In this study, preoperative CAR, in addition to tumor factors and general conditions, was associated with a poor prognosis of CRC after curative resection in older patients. However, only tumor factors were associated with poor prognosis of CRC following curative resection in younger patients. CRP elevation and hypoalbuminemia, which are indicators of chronic inflammation in older people, have been suggested as candidate biomarkers for frailty.^33^ Frailty has recently been reported as a prognostic factor affecting the postoperative outcomes of stage I–III CRC.^5^ In older patients, CAR values calculated from CRP and Alb levels may reflect tumor-induced inflammation and the state of reduced physiological reserve, which was considered the reason why CAR was a sensitive biomarker for the poor prognosis of CRC following curative resection in older patients. CAR reflects low preoperative nutritional status and the immune system, which is the leading cause of poor prognosis after curative resection, and older patients with high CAR on preoperative examination must be identified to provide them with appropriate therapeutic interventions.

Although high CAR levels have been identified as a poor prognostic factor in various carcinomas, appropriate therapeutic interventions to improve CAR levels remain unknown. In older patients, the selection of multidisciplinary treatments, such as nutritional support and aggressive rehabilitation, and avoidance of overly invasive treatments are extremely important. Despite the importance of carefully selecting treatment for older patients, a situation leading to a poor prognosis should be avoided by choosing a treatment strategy that differs from that of younger patients because of age. Yasui et al. reported that the normalization of CAR before and after surgery improved the prognosis of patients with CRC.^30^ Recently, antiinflammatory drug therapy using non-steroidal antiinflammatory drugs, aspirin, histamine-2-receptor agonists, and statins has been shown to suppress host-related inflammatory responses and prevent cancer recurrence.^8,34^ A prospective study would thus be desirable to determine whether CAR reflects the suppression of the inflammatory response by these therapeutic interventions and can be used as a therapeutic indicator.

This study had several limitations. This was a retrospective study, and data were gathered from single centers, which had a limited sample size, particularly for biological markers such as RAS, BRAF, and MSI. More analyses are needed to confirm whether the results are similar to those of the present study after accumulating more cases of these biological markers.

## Conclusions

High preoperative CAR was found to be independently associated with poor prognosis in patients aged ≥ 70 years with stage I–III CRC after curative resection. Preoperative CAR may be a useful assessment tool for predicting the prognosis of older patients who underwent curative resection of nonmetastatic CRC. Further studies investigating treatments to improve CAR are desirable.

### Supplementary Information

Below is the link to the electronic supplementary material.

Supplementary file1 (DOCX 31 KB)

Supplementary file2 (DOCX 29 KB)
