# Supplementary material for: C-Reactive Protein/Albumin Ratio Is an Independent Risk Factor for Recurrence and Survival Following Curative Resection of Stage I–III Colorectal Cancer in Older Patients
Source: Ann Surg Oncol. 2024 Jan 27;31(7):4812–21. doi: 10.1245/s10434-024-14961-2 (PMC11164793; doi:10.1245/s10434-024-14961-2)
Supplement: Supplementary file 1 — Supplementary file1 (DOCX 31 KB) [file 10434_2024_14961_MOESM1_ESM.docx]

Table S1. Univariate and multivariate analyses of prognostic factor for overall survival in patients aged < 70 years

| Variables |  | Univariate |  |  | Multivariate |  |  |
| --- | --- | --- | --- | --- | --- | --- | --- |
|  | N = 329 | HR | 95% CI | *P*-value | HR | 95% CI | *P*-value |
| Male | 207 | 0.857 | 0.38-1.93 | 0.7096 |  |  |  |
| BMI (kg/m^2^) < 25 | 256 | 1.358 | 0.46-3.98 | 0.5763 |  |  |  |
| ASA-PS ≥ 3 | 8 | 2.341 | 0.31-17.4 | 0.4065 |  |  |  |
| HTN (+) | 100 | 2.927 | 1.31-6.54 | **0.0088** | 2.036 | 0.87-4.75 | 0.1004 |
| DM (+) | 51 | 1.059 | 0.36-3.10 | 0.9170 |  |  |  |
| DL (+) | 103 | 1.767 | 0.79-3.95 | 0.1663 |  |  |  |
| Cardiovascular disease (+) | 19 | 2.212 | 0.66-7.42 | 0.1988 |  |  |  |
| Ventilatory disease (+) | 22 | 1.540 | 0.36-6.57 | 0.5593 |  |  |  |
| Medications: antiplatelet or coagulation agents | 20 | 2.247 | 0.67-7.54 | 0.1901 |  |  |  |
| CEA (ng/mL) > 5 | 88 | 2.554 | 1.15-5.69 | **0.0218** | 1.340 | 0.52-3.44 | 0.5430 |
| CA19-9 (U/mL) > 37 | 18 | 7.995 | 3.30-19.3 | **< 0.0001** | 5.317 | 1.77-15.9 | **0.0028** |
| PNI < 47 | 60 | 2.133 | 0.90-5.00 | 0.0816 |  |  |  |
| CAR ≥ 0.03 | 123 | 2.233 | 0.99-5.03 | 0.0525 |  |  |  |
| NLR > 3.0 | 96 | 1.972 | 0.88-4.42 | 0.0990 |  |  |  |
| PLR > 113 | 215 | 0.607 | 0.27-1.38 | 0.2330 |  |  |  |
| Laparoscopic/robot-assisted | 278 | 0.434 | 0.18-1.05 | 0.0636 |  |  |  |
| Operative time (min) > 275 | 187 | 1.822 | 0.76-4.40 | 0.1819 |  |  |  |
| Blood loss (mL) ≥ 60 | 162 | 1.604 | 0.70-3.67 | 0.2635 |  |  |  |
| Intraoperative blood transfusion (+) | 10 | 3.052 | 0.72-13.0 | 0.1311 |  |  |  |
| Tumor localization: Rectum | 174 | 1.614 | 0.71-3.69 | 0.2564 |  |  |  |
| pT ≥ 4 | 17 | 2.771 | 0.93-8.24 | 0.0668 |  |  |  |
| pN (+) | 97 | 2.918 | 1.29-6.61 | **0.0103** | 2.677 | 0.97-7.42 | 0.0584 |
| Histology: other than differentiated carcinoma | 36 | 1.522 | 0.52-4.46 | 0.4451 |  |  |  |
| Vascular invasion (+) | 209 | 5.248 | 1.22-22.4 | **0.0252** | 2.805 | 0.58-13.7 | 0.2015 |
| Budding grade > 1 | 118 | 1.728 | 0.57-5.20 | 0.3313 |  |  |  |
| Postoperative chemotherapy (+) | 134 | 2.796 | 1.15-6.81 | **0.0235** | 0.885 | 0.28-2.81 | 0.8362 |
| Postoperative complications CD≥ 3 (+) | 28 | 1.733 | 0.52-5.82 | 0.3740 |  |  |  |

The variables in bold are statistically significant (*P* < 0.05). Abbreviations: HR, hazard ratio; CI, confidence interval; BMI, body mass index; ASA-PS, American Society of Anesthesiologists Physical Status; HTN, hypertension; DM, diabetes mellitus; DL, dyslipidemia; CEA, carcinoembryonic antigen; CA19-9, carbohydrate antigen 19-9; PNI, prognostic nutrition index; CAR, C-reactive protein/albumin ratio; NLR, neutrophil/lymphocyte ratio; PLR, platelet/lymphocyte ratio; CD, Clavien-Dindo.
