# Supplementary material for: C-Reactive Protein/Albumin Ratio Is an Independent Risk Factor for Recurrence and Survival Following Curative Resection of Stage I–III Colorectal Cancer in Older Patients
Source: Ann Surg Oncol. 2024 Jan 27;31(7):4812–21. doi: 10.1245/s10434-024-14961-2 (PMC11164793; doi:10.1245/s10434-024-14961-2)
Supplement: Supplementary file 2 — Supplementary file2 (DOCX 29 KB) [file 10434_2024_14961_MOESM2_ESM.docx]

Table S2. Univariate and multivariate analyses of prognostic factor for recurrence-free survival in patients aged < 70 years

| Variables |  | Univariate |  |  | Multivariate |  |  |
| --- | --- | --- | --- | --- | --- | --- | --- |
|  | N = 329 | HR | 95% CI | *P*-value | HR | 95% CI | *P*-value |
| Male | 207 | 1.414 | 0.82-2.43 | 0.2086 |  |  |  |
| BMI (kg/m^2^) < 25 | 140 | 1.771 | 0.87-3.59 | 0.1129 |  |  |  |
| ASA-PS ≥ 3 | 8 | 1.500 | 0.37-6.14 | 0.5727 |  |  |  |
| HTN (+) | 100 | 1.645 | 0.99-2.73 | 0.0547 |  |  |  |
| DM (+) | 51 | 1.024 | 0.52-2.01 | 0.9442 |  |  |  |
| DL (+) | 103 | 1.198 | 0.71-2.02 | 0.4987 |  |  |  |
| Cardiovascular disease (+) | 19 | 1.377 | 0.55-3.44 | 0.4928 |  |  |  |
| Ventilatory disease (+) | 22 | 1.008 | 0.37-2.78 | 0.9877 |  |  |  |
| Medications: antiplatelet or coagulation agents | 20 | 0.722 | 0.23-2.31 | 0.5829 |  |  |  |
| CEA (ng/mL) > 5 | 88 | 2.482 | 1.50-4.09 | **0.0004** | 1.663 | 0.97-2.85 | 0.0651 |
| CA19-9 (U/mL) > 37 | 18 | 2.790 | 1.33-5.86 | **0.0068** | 1.670 | 0.75-3.74 | 0.2130 |
| PNI < 47 | 60 | 1.335 | 0.73-2.43 | 0.3445 |  |  |  |
| CAR ≥ 0.03 | 123 | 1.125 | 0.68-1.87 | 0.6487 |  |  |  |
| NLR > 3.0 | 96 | 1.689 | 1.02-2.81 | **0.0436** | 1.293 | 0.75-2.21 | 0.3497 |
| PLR > 113 | 215 | 1.514 | 0.86-2.68 | 0.1544 |  |  |  |
| Laparoscopic/robot-assisted | 278 | 0.526 | 0.29-0.94 | **0.0302** | 1.068 | 0.56-2.04 | 0.8412 |
| Operative time (min) > 275 | 187 | 2.162 | 1.24-3.78 | **0.0068** | 1.282 | 0.68-2.41 | 0.4411 |
| Blood loss (mL) ≥ 60 | 162 | 2.296 | 1.35-3.91 | **0.0022** | 1.755 | 0.97-3.18 | 0.0644 |
| Intra operative blood transfusion (+) | 10 | 1.193 | 0.29-4.89 | 0.8065 |  |  |  |
| Tumor localization: Rectum | 174 | 2.472 | 1.42-4.29 | **0.0013** | 1.863 | 1.02-3.40 | **0.0423** |
| pT ≥ 4 | 17 | 2.516 | 1.14-5.56 | **0.0226** | 1.036 | 0.43-2.48 | 0.9373 |
| pN (+) | 97 | 2.620 | 1.59-4.32 | **0.0002** | 2.147 | 1.06-4.36 | **0.0347** |
| Histology: other than differentiated carcinoma | 36 | 1.102 | 0.50-2.42 | 0.8098 |  |  |  |
| Vascular invasion (+) | 209 | 3.081 | 1.56-6.07 | **0.0011** | 1.694 | 0.80-3.57 | 0.1664 |
| Postoperative chemotherapy (+) | 134 | 2.689 | 1.59-4.53 | **0.0002** | 1.131 | 0.53-2.39 | 0.7475 |
| Postoperative complications CD ≥ 3 (+) | 28 | 0.986 | 0.40-2.46 | 0.9762 |  |  |  |

The variables in bold are statistically significant (*P* < 0.05). Abbreviations: HR, hazard ratio; CI, confidence interval; BMI, body mass index; ASA-PS, American Society of Anesthesiologists Physical Status; HTN, hypertension; DM, diabetes mellitus; DL, dyslipidemia; CEA, carcinoembryonic antigen; CA19-9, carbohydrate antigen 19-9; PNI, prognostic nutrition index; CAR, C-reactive protein/albumin ratio; NLR, neutrophil/lymphocyte ratio; PLR, platelet/lymphocyte ratio; CD, Clavien-Dindo.
